# 

**Published:** 1895-07

**Authors:** 


### CONTENTS FOR JULY, 1895.

ORIGINAL COMMUNICATIONS. PAGE.

Some New Features in the Treatment of Club-Foot. By GREGORY DOYLE, M. D.......... 705

A Few Cases of True Pelvic Cellulitis—A Plea for More Thorough Pelvic Surgery. By ELY VAN DE WARKER, M. D.......... 710

SOCIETY PROCEEDINGS.

Buffalo Academy of Medicine.......... 714

Medical Society of the County of Erie.......... 745

PROGRESS IN MEDICAL SCIENCE.

State Medicine, Public Health, Hygiene and Bacteriology.......... 746

SELECTION.

Cape May as a Health Resort.......... 750

NEW INSTRUMENTS.

The King Thermometer Syringe.......... 752

EDITORIAL.

Our Semi-centennial Anniversary.......... 753

Topics of the Month.......... 754

---

Personal.......... 755

Society Meetings.......... 756

Hospital Notes.......... 758

Medical College Note.......... 759

Book Reviews.......... 760

Literary Note—Miscellany.......... 768

